# Incidence and Prevalence of Epilepsy in Japan: A Retrospective Analysis of Insurance Claims Data of 9,864,278 Insured Persons

**DOI:** 10.2188/jea.JE20220316

**Published:** 2024-02-05

**Authors:** Akemi Kurisu, Aya Sugiyama, Tomoyuki Akita, Ichiro Takumi, Hitoshi Yamamoto, Koji Iida, Junko Tanaka

**Affiliations:** 1 Department of Epidemiology, Infectious Disease Control and Prevention, Graduate School of Biomedical and Health Sciences, Hiroshima University, Hiroshima, Japan; 2 Project Research Center For Epidemiological & Mega-data Analysis of New Research Area, Hiroshima University, Hiroshima, Japan; 3 Department of Neurosurgery, St. Marianna University School of Medicine, Kawasaki, Japan; 4 Department of Pediatrics, St. Marianna University School of Medicine, Kawasaki, Japan; 5 Department of Neurosurgery, Hiroshima University Hospital, Hiroshima, Japan; 6 Epilepsy Center, Hiroshima University Hospital, Hiroshima, Japan

**Keywords:** epilepsy, prevalence, incidence, Japan, claims data

## Abstract

**Introduction:**

The burden of epilepsy is thought to be high but is difficult to measure. Very few studies in Japan have attempted to estimate prevalence and incidence rates of epilepsy in Japan.

**Methods:**

This retrospective cohort study used commercially collected nationwide insurance claims data from a cohort of 10 million persons between 2012 and 2019 among those aged 0 to 74 years. Using the claims data, cases were identified, and incidence and prevalence rates were estimated.

**Results:**

A total of 9,864,278 persons were included. The average age was 34.5 (standard deviation, 18.5) years. A total of 77,312 persons were diagnosed with epilepsy over the 8-year observation period, with a prevalence rate of 6.0 per 1,000 persons with almost no difference by gender. The highest rates were seen among those aged 70–74 years; prevalence rates tended to rise with calendar year (5.4/1,000 in 2012 and 6.0/1,000 in 2019). The incidence rate of epilepsy was 72.1 per 100,000 person-years with slightly higher rates seen among females. Incidence rates were highest at ages less than 12 months (199.8/100,000 person-years), followed by the eldest age group (70–74 years, 179.4/100,000 person-years).

**Conclusion:**

Understanding the magnitude of disease burden is the basis of determining health policies. In this study, the prevalence and incidence of epilepsy in Japan was shown based on the analysis results of a large-scale general population insurance claims data covering all over Japan.

## INTRODUCTION

Epilepsy is a neurological disorder affecting an estimated 50 million persons world-wide.^1^ The disease occurs in all age groups from infants to the elderly. Meta analyses of worldwide rates show large variations due to study design differences and differing case definitions.^2^ Recent estimates indicate that the prevalence rate is reported to be 4–8 per 1,000 persons,^3^^,^^4^ while the incidence rate is approximately 45–49 per 100,000 population per year in high-income countries. Rates are higher in low- and middle-income countries.^2^^,^^5^ As there have been few epidemiological reports on Japanese national rates of epilepsy, the burden of epilepsy among the population in Japan remains an open research question. Understanding basic epidemiological information, such as prevalence and incidence, is important as a basis for constructing effective disease control measures. “Patient survey” is one of the statistical surveys conducted by the government every 3 years in Japan to estimate the number of patients for all diseases. However, the number of cases based on the patient survey is likely underestimated. Even though patients may be diagnosed with several diseases, the patient survey makes estimates based on only one main disease for each patient. Moreover, the number of patients with chronic diseases may be underestimated, as only those whose interval visit is under 30 days are included (this criterion has been changed to 98 days since 2020). Furthermore, the patient survey cannot provide estimates of incidence. The purpose of this study was to determine the prevalence and incidence of epilepsy in Japan using a large population-based data set.

## METHODS

This retrospective cohort study used monthly medical claims data collected in the Japan Medical Data Center (JMDC) database (Japan Medical Data Center, Tokyo, Japan) for 8 years from January 2012 through December 2019. The JMDC system collects information from Japanese health insurance organizations that provide health insurance to employees of large Japanese companies and their dependents.^6^ Persons employed by small- and medium-sized companies, National Health Insurance subscribers, or welfare recipients were not included. Also, persons certified as having a disability due to epilepsy between ages 64–74 were excluded, as they are eligible for alternative medical benefits. Insured persons could be enrolled for multiple years over the course of the observation period. Persons less than 74 years of age were eligible for the study, as that was the upper limit of persons insured by this insurance association.

**Figure 1.**
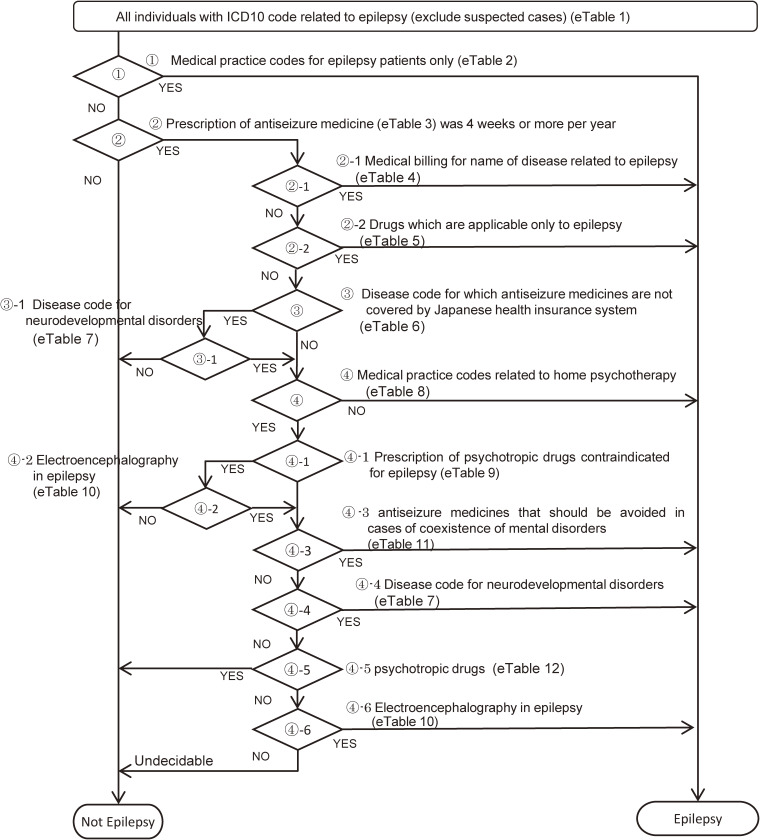
Decision tree for identifying those with epilepsy

Cases were identified using diagnosis code (International Classification of Diseases, 10^th^ revision [ICD-10]: G40 Epilepsy, G41 Status epilepticus) or a combination of epilepsy-related injury/illness codes and medications. This definition does not include febrile convulsions (ICD10: R56.0). The decision tree was designed to only select those with epilepsy, excluding suspected cases, and included a verification step where results were checked on a month-by-month basis to optimize the algorithm (Figure 1, eTable 1, eTable 2, eTable 3, eTable 4, eTable 5, eTable 6, eTable 7, eTable 8, eTable 9, eTable 10, eTable 11, and eTable 12). This decision tree was developed through discussion with multiple expert physicians for epilepsy belonging to the Ministry of Health, Labour and Welfare research group.

After identifying the unique (non-duplicate) target population and cases, the prevalence of epilepsy by sex and age group was calculated as the ratio of cases to eligible health insurance association members. Prevalence was calculated by year as well as for the 8-year period from January 2012 through December 2019. Prefecture-specific prevalence rates could not be calculated, as prefecture information was not available on the supplied records. The 95% confidence intervals (CIs) were estimated using Wald’s method. The prevalence of epilepsy were compared by calendar year using the Cochran-Armitage test.

The incidence of epilepsy was calculated as the rate of new cases over the 8-year follow-up period (January 2012 through December 2019) divided by the person-years at risk. Prior claims were available back to 2005 (earliest), and persons known to have been diagnosed with epilepsy prior to January 2012 (based on “date of start treatment”) were excluded from the analysis. Cases diagnosed within 1 year of joining JMDC were censored because the possibility that the diagnosis was due to a change in insurance. The 95% CIs were estimated using the Poisson distribution for incidence as follows:

$$\begin{array}{rl} & \text{95\% lower limit of incidence rate}=\chi_{2r}^{2}(0.975)/(2T)\\ & \text{95\% upper limit of incidence rate}=\chi_{2r+2}^{2}(0.025)/(2T) \end{array}$$

where *r* and *T* are the number of new cases detected during the observational period and the total observed person-years, respectively. $\chi_{2r}^{2}(0.975)$ was the upper 97.5% point of a *χ*^2^ distribution with 2*r* degrees of freedom (df) and $\chi_{2r+2}^{2}(0.025)$ the upper 2.5% point of a *χ*^2^ distribution with 2*r* + 2 df.

The incidence rate at ages less than 12 months and the eldest age group (70–74 years) were compared to the other age groups using Z test adjusted using the Bonferroni test. Z-variable is obtained as follows:

$$Z=\frac{I_{1}-I_{2}}{\sqrt{\left(r_{1}+r_{2})/(T_{1}T_{2}\right)}}$$

where *I*_1_ and *I*_2_ denoted the incidence rates, *r*_1_ and *r*_2_ denoted the number of new cases of epilepsy, and *T*_1_ and *T*_2_ were the observation periods (person-years). The crude *P*-value was obtained by calculating the upper probability of Z under the normal distribution and adjusted using the Bonferroni method.

This study was conducted after obtaining permission from the Ethical Review Committee for Epidemiological Research at Hiroshima University for ethical review of medical research (epidemiology) involving human subjects (Permission No.: E-2298). The claims data used for the analysis is existing information after anonymization. Individuals cannot be identified and no disadvantage to any subjects is anticipated.

## RESULTS

A total of 9,864,278 eligible individuals were identified over the 8-year period from January 2012 through December 2019. Insured subjects could be enrolled for multiple years over the course of the observation period. The number of eligible subjects enrolled in a 1-year period ranged from 1,785,253 persons in 2012 to 7,442,394 persons in 2019 (Table 1).

**Table 1.** Count of eligible persons included in the study based on the JMDC Claims Database, by year

Year	Number of health insurance subscribers
2012	1,785,253
2013	2,824,800
2014	2,979,938
2015	4,174,628
2016	5,202,333
2017	6,199,167
2018	7,236,266
2019	7,442,394
Total^*^	9,864,278

JMDC, the Japan Medical Data Center.

^*^Excluding duplicates.

**Figure 2.**
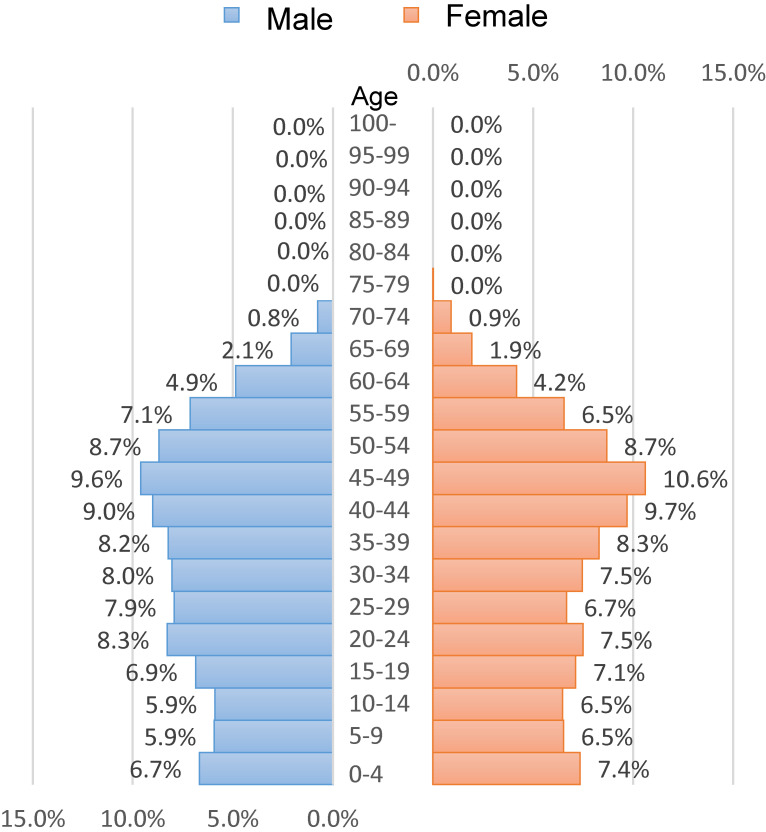
Age distribution by sex of the study population in 2019 (*N* = 7,442,394)

The average age of 7,442,394 persons in 2019 was 34.5 (standard deviation, 18.5) years, and males comprised 53.7% of the sample. The distribution of persons by age and sex is shown in Figure 2. For each sex, 45–49 years old was the most common 5-year age group.

A total of 77,312 persons were diagnosed with epilepsy over the follow-up period. The prevalence of epilepsy was 6.0 per 1,000 population with almost no difference by gender (6.1 for males and 5.8 for females in 2019). The secular change in epilepsy prevalence from 2012 to 2019 was a slight upward trend (5.4/1,000 population in 2012 rising to 6.0/1,000 in 2019) (trend *P* < 0.0001). This upward trend was seen in both males (5.4–6.1/1,000) and females (5.4–5.8/1,000) (both trend *P* < 0.0001) (Table 2).

**Table 2.** Count persons, prevalent cases, and prevalence rates, by calendar year and sex

Year	Total				Male				Female			

	Persons (*n*)	Prevalent Cases (*n*)	Prevalence Rate per 1,000	Trend P^*^	Persons (*n*)	Prevalent Cases (*n*)	Prevalence Rate per 1,000	Trend P^*^	Persons (*n*)	Prevalent Cases (*n*)	Prevalence Rate per 1,000	Trend P^*^
2012	1,785,253	9,705	5.4	*P* < 0.0001	1,001,565	5,456	5.4	*P* < 0.0001	783,688	4,249	5.4	*P* < 0.0001
2013	2,824,800	15,319	5.4		1,568,664	8,476	5.4		1,256,136	6,843	5.4	
2014	2,979,938	16,355	5.5		1,656,390	9,076	5.5		1,323,548	7,279	5.5	
2015	4,174,628	23,436	5.6		2,302,577	12,991	5.6		1,872,051	10,445	5.6	
2016	5,202,333	29,950	5.8		2,867,978	16,645	5.8		2,334,355	13,305	5.7	
2017	6,199,167	36,129	5.8		3,372,022	19,988	5.9		2,827,145	16,141	5.7	
2018	7,236,266	42,736	5.9		3,897,629	23,481	6		3,338,637	19,255	5.8	
2019	7,442,394	44,568	6		3,995,265	24,474	6.1		3,447,129	20,094	5.8	

^*^Cochran-Armitage test.

**Figure 3.**
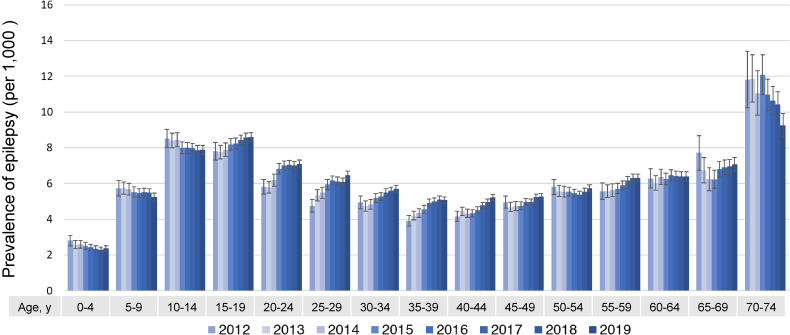
Prevalence of epilepsy by year and age group

By age group, the prevalence of epilepsy in the 70–74 age group was the highest compared to other age groups (9.2/1,000 in 2019), followed by those in their teens (15–19 years: 8.6/1,000; 10–14 years: 7.9/1,000 in 2019) (Figure 3).

**Figure 4.**
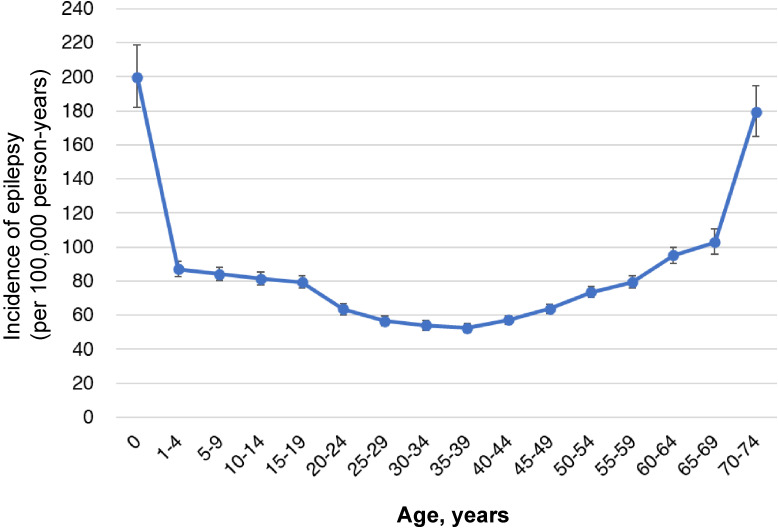
Incidence rate of epilepsy by age (with 95% CIs). CI, confidence interval.

Among the 77,312 subjects diagnosed with epilepsy, 47,387 were diagnosed prior to January 2012. Another 5,925 subjects were diagnosed with epilepsy within 1 year of joining the health insurance association and were censored from the incidence calculation. Therefore, there were 24,000 incident cases among 33,299,234 eligible person-years of follow-up from 2012–2019 resulting in an incidence rate of 72.1 per 100,000 person-years (95% CI, 71.2–73.0/100,000 person-years). The incident rate in females (73.7; 95% CI, 72.3–75.1/100,000 PY) was slightly higher than that observed in males (70.7; 95% CI, 69.5–72.0/100,000 PY) (Table 3). By age group, the incidence rate was highest at ages less than 12 months (199.8; 95% CI, 182.1–218.7/100,000), followed by the eldest age group (70–74 years) at rates of 179.4/100,000 person-years (95% CI, 164.9–194.7/100,000 person-years), which was significantly higher than the other age groups (*P* < 0.0001). A U-shaped curve was observed (Figure 4). The age-specific complication rates of stroke (ICD-10: I63, I69) and Alzheimer’s disease (ICD-10: G30) in epilepsy patients were shown in Table 4. Among epileptic patients aged 70–74 years, 24.1% had stroke, 15.1% had post-stroke sequelae, and 12.8% had Alzheimer’s disease.

**Table 3.** Incidence of epilepsy (per 100,000 person-years) observed in the cohort over the period 2012–2019, by sex

	Persons (*n*)	Person-Years	Number of incident cases	Incidence Rate (per 100,000 person-years)	95% CI
Total	9,816,891	33,299,234	24,000	72.1	71.2–73.0
Male	5,139,227	18,401,137	13,018	70.7	69.5–72.0
Female	4,677,664	14,898,098	10,982	73.7	72.3–75.1

**Table 4.** The age-specific complication rates of stroke (ICD10: I63, I69) and Alzheimer’s disease (ICD10: G30) in epilepsy patients

ICD10	ICD-10 Disease name	Number of epilepsy patients by age group																	

		0–9 years *N* = 3,881		10–19 years *N* = 3,658		20–29 years *N* = 2,725		30–39 years *N* = 2,890		40–49 years *N* = 4,043		50–59 years *N* = 3,950		60–69 years *N* = 2,284		70–74 years *N* = 569		Total *N* = 24,000	
I63	Stroke	0	0%	0	0%	0	0%	0	0%	0	0%	0	0%	365	15.9%	137	24.1%	502	2.1%
I69	Post-stroke sequelae	0	0%	0	0%	0	0%	0	0%	0	0%	0	0%	265	11.6%	86	15.1%	351	1.5%
G30	Alzheimer’s disease	0	0%	0	0%	0	0%	0	0%	0	0%	0	0%	0	0%	73	12.8%	73	1.4%

ICD-10, International Classification of Diseases, 10^th^ revision.

## DISCUSSION

Patients with epilepsy worldwide were estimated to be 45.9 million in 2016 using a simulation model, and the age-standardized prevalence in Japan was 130 to <180 per 100,000 population, which was lower than that of the world.^7^ Understanding the magnitude of disease burden is the basis of determining health policies. In this study, we estimated the prevalence and incidence of epilepsy in Japan using a large-scale claims-based database consisting of approximately 10 million people (2012–2019) aged between 0 and 74 years covered all over Japan. In this study, the prevalence of epilepsy (2012–2019) was calculated to be 5.4–6.0 per 1,000 population. This result is consistent with reports on epilepsy prevalence in Asia: 6 per 1,000 population.^8^ The prevalence in Europe is at the same level: 4.5–5.0 in children and 6.0–7.0 in adults per 1,000 population,^9^ but it is higher in Africa and South America (15 per 1,000 in sub Saharan Africa and 18 per 1,000 in Latin America).^8^ A population-based study from Japan surveying residents aged 40 and over reported a prevalence of epilepsy of 6.9 per 1,000.^10^ Another population-based study of children aged younger than 13 years reported that the prevalence of epilepsy, including febrile convulsions, was 8.8 per 1,000, and the prevalence of epilepsy, excluding febrile convulsions, was 5.3 per 1,000.^11^ However, both were reports of surveys in limited areas. A nationwide statistical survey conducted in 2020 in Japan reported a prevalence of 3.3 per 1,000 population (3.7 for male, 3.0 for female), which was lower than the results of this study.^12^ The number of patients from the patient survey may have been underestimated, as mentioned above. The prevalence of epilepsy was nearly the same for both sexes, and this result was consistent with previous reports.^2^^,^^9^^,^^10^

The prevalence of epilepsy by age group in developed countries is known to be highest in the elderly, because symptomatic epilepsy associated with stroke or Alzheimer’s disease increases with age.^9^^,^^13^^,^^14^ This study also showed a high comorbidity of stroke and Alzheimer’s disease among subjects who were 70–74 years old. In the present study, the prevalence of epilepsy in the age group of 70–74 years was the highest compared to other age groups, followed by those in their teens. The influence of benign childhood epilepsy with a short disease duration may be one of the reasons why the prevalence of epilepsy in teenagers is higher than in other age groups.

The incidence of epilepsy in females was slightly higher than that in males. By age group, the incidence of epilepsy was highest at less than 12 months of age, followed by the age group 70–74 years. These age-dependent incidence trends were similar to previously reported studies.^15^^–^^18^

This is the first report to estimate the incidence of epilepsy based on large-scale insurance claims in Japan. These estimates, based on a large representative cohort covering subjects aged 0–74 in all regions of Japan, are important, as they help determine public health needs, such as building a medical system for epilepsy patients through cooperation between general practitioners and specialists, as well as enhancing support systems for families in Japan. The incidence calculated by this study is about 1.5 times higher than the incidence of 45–49/100,000 person-years obtained from previous meta-analysis reports in developed countries.^2^^,^^5^ Since insurance claims are documents for medical billing, they may be artificially inflated, so the possibility of overestimation cannot be ruled out. However, we guarded against this bias by taking the following measures: (1) comparing diagnoses in chronological order, (2) adopting information that includes epilepsy guidance fees, and (3) collecting information on antiseizure medicine drug administration. On the other hand, it is also possible that past reports were underestimated due to selection bias. This study suggests that the incidence of epilepsy may be higher than conventionally recognized, suggesting the need to further evaluation to confirm that support system for epilepsy patients and their families are sufficient.

### Limitations

There were several study limitations that may reduce the generalizability of its conclusions. While the cohort included nearly 10 million persons, it was limited to those employed by large companies and their dependents younger than 75 years old, which did not include employees of small- and medium-sized companies, National Health Insurance subscribers, or welfare recipients. This suggests that the representativeness of this population for the Japanese population is limited but should provide important initial results based on Japanese population data. Other exclusions included persons certified as having a disability due to epilepsy between ages 64–74, as they are eligible for alternative medical benefits. As a result, there is a possibility that the number of epilepsy patients in the 64–74 years age group could be slightly underestimated. As for the definition of epilepsy, a validation study was not performed, though the decision tree of this study was developed with multiple expert physicians. In addition, analysis of the type of epilepsy was not possible because detailed classification of epilepsy was often not listed in the medical claims. However, even with those limitations, there have been very few epidemiological studies reporting the burden of epilepsy in a large general population covering all of Japan, meaning that the results of this study should be important for evaluating disease control policies for epilepsy in Japan. To understand the prevalence and incidence of epilepsy among all ages, including those aged 75 years and older, analysis using National Database of Health Insurance Claims and Specific Health Checkups of Japan (NDB) is desired in the future.

## SUPPLEMENTARY MATERIAL

eTable 1. List of ICD-10 codes related to epilepsy

eTable 2. List of Japan’s original code of medical billing for medical practice for epilepsy patients

eTable 3. List of antiseizure medicine

eTable 4. List of Japan’s original code of medical billing for name of disease related to epilepsy

eTable 5. List of the codes specified by the Ministry of Health, Labor and Welfare Japan for drugs which are applicable only to epilepsy

eTable 6. List of disease codes for which antiseizure medicine are not covered by Japanese health insurance system

eTable 7. List of disease name codes for neurodevelopmental disorders

eTable 8. List of medical practice codes related to home psychotherapy for outpatients

eTable 9. List of drug codes for psychotropic drugs contraindicated for epilepsy

eTable 10. List of medical practice codes for electroencephalography in epilepsy

eTable 11. List of drug codes of antiseizure medicine that should be avoided in cases of coexistence of mental disorders

eTable 12. List of psychotropic drugs
