# Supplementary material for: Incidence and Prevalence of Epilepsy in Japan: A Retrospective Analysis of Insurance Claims Data of 9,864,278 Insured Persons
Source: J Epidemiol. 2024 Feb 5;34(2):70–5. doi: 10.2188/jea.JE20220316 (PMC10751190; doi:10.2188/jea.JE20220316)
Supplement: Supplementary file 1 [file je-34-070-s001.pdf]

eTable 1. List of ICD-10 codes related to epilepsy

**ICD-10**	
**G40 Epilepsy**	
	G40.0
	G40.1
	G40.2
	G40.3
	G40.4
	G40.5
	G40.6
	G40.7
	G40.8
	G40.9
**G41 Epilepsy status (condition)**	
	G41.0
	G41.1
	G41.2
	G41.8
	G41.9
**F80 Specific developmental disorders of conversation and language**	
	F80.3
**G09 Secondary / sequelae of central nervous system inflammatory disease**	
**T90 Secondary / sequelae of head injury**	
	T90.5

ICD-10, International Classification of Diseases, 10th revision.

eTable 2. List of Japan's original code of medical billing for medical practice for epilepsy patients

Medical practice	Japan's original code for medical practice
**B001_6 Epilepsy guidance fee**	113002850
**C110_3 Home vagus nerve electrical stimulation treatment guidance management fee**	114021610

eTable 3. List of antiseizure medicine

Drug classification	The Anatomical Therapeutic Chemical (ATC) Classification Code
**1131 Phenasemide preparations**	N03AX13
**1132 Hydantoin preparations**	N03AB01
	N03AB02
**1133 Oxazolidine preparations**	N03AC02
**1134 Barbituric acid preparation**	N03AA02
**1135 Primidone preparations**	N03AA03
**1136 Aminobutyric acid preparations**	N03AG03
**1137 Sulfonamide preparations**	N03AX03
**1139 Other antiseizure medicine**	N03AB52
	N03AD01
	N03AE01
	N03AE01
	N03AF03
	N03AG01
	N03AG04
	N03AX09
	N03AX11
	N03AX14
	N03AX14
	N03AX15
	N03AX17
	N03AX18
	N03AX22
	N05BA09
	N05CD02
	N05CM11
**2134 Carbonic anhydrase inhibitors**	S01EC01

eTable 4. List of Japan's original code of medical billing for name of disease related to epilepsy

Name of disease	Japan's original code for medical billing
Localized/focal epilepsy	3455005
Focal sensory seizures	8834773
Focal epilepsy	3455010
Jacksonian eilepsy	3455001
Autonomic nervous system epilepsy	3458007
Focal somatosensory seizure	3458017
Delayed epilepsy	3458024
Auditory seizures	3458019
Epilepsy simple partial epilepsy	8846872
Infant epilepsy with migratory focal seizure	8849555
Medial temporal lobe epilepsy with hippocampal sclerosis	8849521
Psychomotor seizures	3454005
Frontal lobe epilepsy	3451019
Temporal lobe epilepsy	3454009
Epileptic Automatism	8837937
Epilepsy Complex Partial Seizures	8846873
Partial Epilepsy	8839840
Absence	3450002
Tonic-clonic seizures	3451012
Childhood Absence Seizure	8834779
Progressive myoclonus epilepsy	8834977
Juvenile Absence Epilepsy	8835246
Juvenile Myoclonic Epilepsy	8835263
Formal absence seizures	3450004
West syndrome	3456001
Ohtawara syndrome	8849520
Early myoclonic encephalopathy	8849542
Point-blank epilepsy	3456004
Dravet syndrome	8849546
Infant Severe Myoclonic Epilepsy	8847543
Myoclonic absence epilepsy	8849552
Epilepsy with myoclonic weakness seizures	8849553
Lennox-Gastaut Syndrome	8841171
Unilateral convulsive hemiplegic epilepsy syndrome	8840001
Refractory epilepsy	3458025
Post-stroke epilepsy	3458023
Landau-Kleffner syndrome	8840927
Post-Encephalitis Epilepsy	8846326
Traumatic Epilepsy	9070002
Early trauma epilepsy	9070012

eTable 5. List of the codes specified by the Ministry of Health, Labor and Welfare Japan for drugs which are applicable only to epilepsy

Japan's Ministry of Health, Labour and Welfare code for drugs
1131001A1038
1131001F1035
1132001A1035
1132002B1019
1132002B1019
1132002B1019
1132002F1010
1132002F1010
1132002F1010
1132002F2017
1132002F2017
1132002F2017
1132002B1019
1133002B1032
1137001F1039
1137001F2035
1139001B1031
1139001Q1042
1139012F1023
1139012F2020
1139013B1026
1139008C1020
1139008F1027
1139008F1035
1139008F2023
1139008F2031
1139008F3020
1139008F3038
1139010F1024
1139010F2020
1139010R1020
1139005B1013
1139005B1048
1139005F1015
1139005F1023
1139005B1013
1139005B1030
1139005F1015
1139005F1015
1139005F1031
1139011M1028
1139011R1025
1139011R2021
1139015F1027
1139015F2023
1139015R1023
1139014F1022
1139014F2029
1126001X1014
1126002X1019

eTable 6. List of disease codes for which antiseizure medicine are not covered by Japanese health insurance system

ICD-10	Japan's original code for medical billing
**B02**	8836919
	531012
**E10－E14**	8845056
	8845085
	2505011
	8848768
**G50**	3501002
	3501010
	8834074
	8834075
	8834076
	8834077
	8834078
	8834079
	8834702
	8835405
	3501025
	3501010
**G51**	8832082
	8832083
**G64**	8846220
	8849550
**G96**	8849545
	8849544
**G98**	8847489
**R52**	8847738
	7998003
	8847739
	8844739
	7890030
	8847802
	8847821
**M79**	8836065
**G24**	8841457
	8841454
	8842320
	8842321
	8848454
	8841453
	8841455
	7235010
	8849222
	8833474
	8841404
	8841405
	8841452
	3338005
	8841345
	8848693
	8848707
	8848711
	8848731
	8835214
	8841450
**G25**	3335004
	3335007
	3335009
	8834822
	8836844
	8839823
	8841412
	8841413
	8845029
**G43**	3461002
	8849148
	3460002
	8840019
	8849085
	8849143
	8849147
	8849178
	8849179
	8849180
	8849186
	8849306
	8835221
	8849199
	8832031
	8849198
	3468001
	8840640
	8849461
	3469004
	8849213

ICD-10, International Classification of Diseases, 10th revision.

eTable 7. List of disease name codes for neurodevelopmental disorders

ICD-10	Japan's original code for medical billing
**F80**	8838167
	8838964
	8840884
	8848327
	8839413
	7843001
	8831489
	8835369
	8836301
	8840927
	8831346
	3153007
	7845002
F81	3150002
	8838169
	8834868
	8838168
	3108001
	8832912
	8838963
	3150001
	8831871
	8838171
	8838966
	8831870
	8831872
F82	3154007
	3488016
	8830803
	8838962
F90	3073008
	8845700
	8834784
	8836993
F91	8845643
	8845724
	8836883
	8845670
	8838999
	3129003
	8845675
	8845696
F92	8845739
	3123001
	8833333
F93	8839903
	8838255
	8831243
	3139001
F94	8836121
	8843354
	8830159
	8834364
	8834781

ICD-10, International Classification of Diseases, 10th revision.

eTable 8. List of medical practice codes related to home psychotherapy for outpatients

Japan's original code for medical billing
180055510
180020410
180012210
180031010
180007250
180031210
180049130
180049230
180049330
180049830
180049930
180055970
180056030
180058530
188003310
188003410
188003670
188005110
188011110

eTable 9. List of drug codes for psychotropic drugs contraindicated for epileps

Japan's Ministry of Health, Labour and Welfare code for drugs
1179017F1013
1179017F1013
1179017F1072
1179017F2010
1179017F2010
1179017F2079
1179017F1056
1179017F2052
1179008F1022
1179008F2029
1179008F1014
1179008F1014
1179008F2010
1179008F2010
1179008F3068
1179023F1035
1179023F2023
1179023F3020
1179049F1021
1179049F2028

eTable 10. List of medical practice codes for electroencephalography in epilepsy

Japan's original code for medical billing
160075570
160075610
160200510
160075750
160075850
160075950
160076050
160075310
160170610
160207510
160187010
160218410
160175810

eTable 11. List of drug codes of antiseizure medicine that should be avoided in cases of coexistence of mental disorders

The Anatomical Therapeutic Chemical (ATC) Classification Code	Japan's Ministry of Health, Labour and Welfare code for drugs
N03AA02	1125003B2015
	1125003B2015
	1125003B2031
	1125003B2139
	1125003B2163
	1125003X1016
	1125003X1016
	1125003X1016
	1125004F1023
	1125004S1030
N03AA03	1135002C1059
	1135002F1055
N03AD01	1139001B1031
	1139001Q1042
N03AX11	1139008C1020
	1139008F1027
	1139008F1035
	1139008F2023
	1139008F2031
	1139008F3020
	1139008F3038
N03AX14	1139010F1024
	1139010F2020
	1139010R1020
N03AX15	1139005B1013
	1139005B1048
	1139005F1015
	1139005F1023
	1139005B1013
	1139005B1030
	1139005F1015
	1139005F1015
	1139005F1031

eTable 12. List of psychotropic drugs

The Anatomical Therapeutic Chemical (ATC) Classification Code
N05BE
N05AB02
N06AA04
N06AA07
N06AA17
N06AA10
N05BB01
N05AD05
N05BA21
N05AD01
N05AX11
N05BA19
N05AD
N05AD06
N05AX
N05AX08
N06AB08
N06AB05
N05AH04
N05AH03
